# Epidemiological and Diagnostic Aspects of Bladder Bilharziomas in the Urology Department of Idrissa Pouye General Hospital (HOGIP)

**DOI:** 10.1155/2021/4536381

**Published:** 2021-03-25

**Authors:** Oumar Gaye, Mohamed Jalloh, Ngor M Thiam, Medina Ndoye, Khadidiatou Dansokho, Youssef Bellamine, Saint C. N Kouka, Cherif Dial, Mouhamadou M Mbodji, Ayun Cassell, Issa Labou, Lamine Niang, Serigne Gueye

**Affiliations:** ^1^Urology-andrology Department, Hôpital General Idrissa Pouye, Dakar, Senegal; ^2^Pathological Anatomy Laboratory, Hôpital General Idrissa Pouye, Dakar, Senegal; ^3^Urology-andrology Department, UFR Santé Université de Thies, Dakar, Senegal

## Abstract

**Objective:**

The aim of our study is to assess the diagnostic aspects of bladder bilharzioma in the Urology Department of Idrissa Pouye General Hospital (Senegal).

**Materials and Methods:**

It is a descriptive study from January 2013 to December 2018. The patients included in the study were those who had anatomopathological examination of bladder biopsy that showed a schistosomiasis pseudotumor of the bladder. The variables studied were sociodemographic, clinical symptoms, imaging findings, histology, and treatment. The data have been saved and analyzed by the 2013 Excel software.

**Results:**

Thirteen patients were included in our study. The average age was 27 ± 12.1 years. Sex ratio was 1.6. The majority of the patients were from the northern part of Senegal. Hematuria was the main symptom for all the patients. Cystoscopy was performed for all the patients and showed 5 granulomas and 8 fibrocalcic polyps. A transurethral resection of the bladder was performed, and treatment with praziquantel (40 mg/kg of bodyweight) has been carried out. One patient presented precancerous lesions such as metaplasia and dysplasia of the bladder mucosa. After a median follow-up of 40 months (6–57 months), ten patients had a favorable clinical and endoscopic outcome.

**Conclusion:**

Granulomas and fibrocalcic polyps of the bladder mucosa are, respectively, confused with squamous cell carcinoma and bladder lithiasis in endemic areas of schistosomiasis. Good cystoscopy interpretation can provide the diagnosis of bladder bilharzioma and start the treatment.

## 1. Introduction

*Schistosoma haematobium* bilharziasis is responsible for urogenital bilharziasis. It reaches a hundred million people in sub-Saharan Africa [1]. In Senegal, it prevails in all regions with a prevalence reaching more than 50% in certain areas [2, 3]. Infection of humans with schistosomes occurs through contact with water contaminated with furcocercariae which actively penetrate through the skin. The breeding grounds for the disease are stagnant water, the banks of rivers, the banks of lakes, and irrigation canals. Children are the most affected age group [2, 4]. Fishermen, cultivators, rice farmers, and workers who maintain irrigation canals are the most exposed adults. Women, because of their daily frequentation in places at risk for household chores, are more affected than men [4].

As soon as they penetrate through the skin, the schistosomules reach the intrahepatic portal venules by circulatory route. They will continue their sexual development and maturation towards the venous plexuses of the bladder and other pelvic organs [5]. The involvement of the various urogenital organs varies according to the richness of their vascularization. This is how the bladder, seminal vesicles, and the end of the ureters are more affected by the disease, thanks to their rich vascular supply [6].

In the bladder plexus, the female leaves the male to enter the submucosa where she begins to lay eggs. Some eggs will be eliminated by the urine, but many of them will remain blocked in the bladder wall or will be embolized at a distance [4, 7].

The eggs blocked in the tissue will determine the formation of a bilharzian granuloma. Indeed, these eggs secrete an antigen that triggers a cell-mediated immune response. The cell infiltrate is characterized by the presence of eosinophils, histiocytes, fibroblasts, epithelioid, and giant cells [6].

Bladder bilharziomas can manifest clinically as hematuria, lower urinary tract symptoms, or acute retention of urine [8–10].

El-Badawi described three types of bladder bilharzioma: granulomatous polyps, fibrocalcic polyps, and villous polyps [8].

The granulomatous polyp and the fibrocalcic polyp pose, respectively, the problem of differential diagnosis with squamous cell carcinoma of the bladder and bladder stones in the area of bilharzian endemia. Nevertheless, a good exploration with cystoscopy makes it possible to differentiate them from the latter and to propose an adequate treatment. Villous polyps are more difficult to diagnose and require histological analysis [8].

The treatment of bladder bilharziomas is related to antibilharzian treatment. Praziquantel at a dose of 40 mg/kg is recommended. Transurethral resection of the bladder and bladder biopsy are indicated for residual masses or when the endoscopic diagnosis of bilharzioma is not obvious [6].

The objective of our study was to describe the diagnostic aspects of bladder bilharziomas in our center.

## 2. Materials and Methods

This is a descriptive study from January 2013 to December 2018. The patients included were those who underwent transurethral resection of the bladder indicated before a bladder tumor and whose results of the anatomopathological examination of the shavings of resection concluded in a bilharzian pseudotumor of the bladder. The parameters studied were age, sex, geographic origin, clinical signs, type, location and number of bilharziomas, results of anatomopathological examination of the resection chips, treatment, and course. The data were recorded and analyzed by Excel 2013 software.

## 3. Results

**Figure 1:**
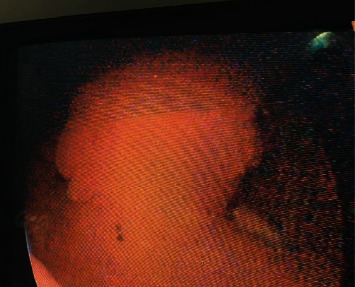
Polyp sitting around the left ureteral meatus viewed with cystoscopy.

**Figure 2:**
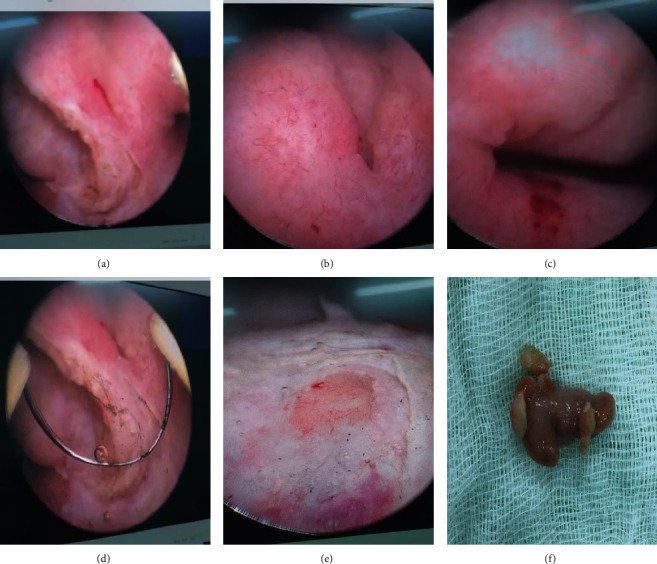
Polyp granulomatous sitting around the left ureteral meatus. (a) Granulomatous polyp developed on left ureteral meatus. (b) Left ureteral meatus. (c) Granulomatous polyp and its implantation base. (d) Resection of the granulomatous polyp. (e) Aspect of the left ureteral meatus after resection. (f) Resected granulomatous polyp.

**Figure 3:**
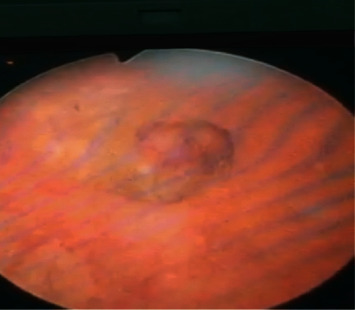
Fibrocalcic polyp located at the level of the bladder dome visible on cystoscopy.

**Figure 4:**
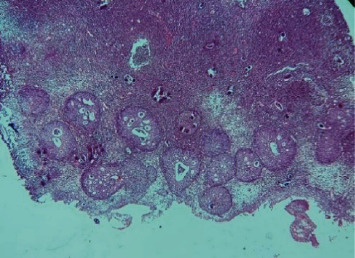
Giant cells phagocytizing eggs of viable bilharzia. Hematoxylin and eosin x 100 (image of Professor Dial, head of the pathological anatomy laboratory of HOGIP).

**Figure 5:**
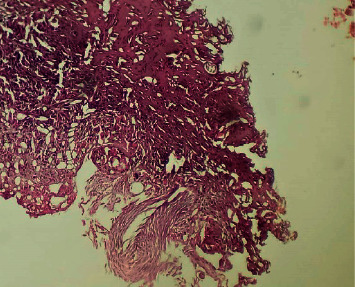
Granulomatous inflammatory reaction and not viable bilharzia egg. Hematoxylin and eosin x 100 (image of Professor Dial, head of the pathological anatomy laboratory of HOGIP).

**Figure 6:**
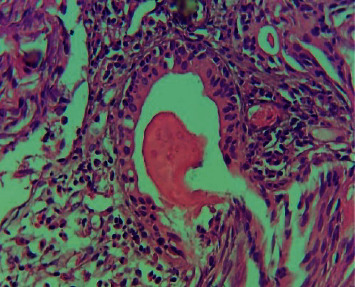
Dysplastic lesion associated with granulomatous inflammatory reaction due to *Schistosoma haematobium*. Hematoxylin and eosin x 100 (image of Professor Dial, head of the pathological anatomy laboratory of HOGIP).

This study involved 13 patients. The mean age of the patients was 27 ± 12.1 years. The sex ratio was 1.6. Ten of our patients were from northern Senegal in the Senegal River region. A notion of swimming in fresh water had been clarified in eleven of the patients. Hematuria was the finding in all patients and was associated with dysuria in two patients (Table 1). A urine pellet was produced in two of our patients, one of whom isolated bilharzia eggs. The cystoscopy performed in all the patients objectified five granulomatous polyps (38.5%) and eight fibrocalcic polyps (61.5%). It also showed lesions of semolina vesical bilharziasis associated in the five granulomatous polyps (Figures 1 and 2) and in four of the fibrocalcic polyps (Figure 3). The granulomatous polyps were unique in three patients and were located mainly in the perimeatic regions. The fibrocalcic polyps were unique in six patients and were mainly located in the bladder dome (Table 2). An ultrasound of the urinary tree was performed in 2 patients and showed focal parietal thickening of the bladder without affecting the upper urinary tract. A URO-computed tomography (URO-CT) was performed in 2 patients and objectified a bladder tumor (Figure 3). Transurethral resection of the bladder tumor (TURBT) associated with medical treatment with praziquantel (40 mg/kg as a single dose) had been performed in all our patients. Pathological examination of the resection shavings showed an epitheliogigantocellular and polynuclear inflammatory eosinophilic granulomatous inflammatory reaction around bilharzian eggs belonging to the species *Schistosoma haematobium* (Figures 4 and 5). Precancerous lesions such as metaplasia and dysplasia of the bladder mucosa were objectified in one of our patients (Figure 6). After a median follow-up of 40 months (6–57 months), three patients were lost to follow-up. Good clinical and endoscopic improvement was noted in nine patients with disappearance of hematuria and cystoscopy images in nine patients. A recurrence in one patient required a second RTUV whose histology concluded with a bilharzioma.

## 4. Discussion

Bladder bilharziomas are inflammatory pseudotumors secondary to *Schistosoma haematobium* infection and results from chronic inflammation. The presence of such tumor poses a problem of differential diagnosis. Generally, the confirmation of the diagnosis is performed after TURBT. In spite of the controversies regarding the role of schistosomiasis as a risk factor for bladder cancer, any patient with hematuria or the diagnosis of bladder mass will be investigated for a history of bladder schistosomiasis.

Ten of our patients were from northern Senegal living next to Senegal river. This area is known for its high prevalence of bladder schistosomiasis (53%) [3, 11].

The mean age of our patients was 27 years which is in accordance to the higher prevalence of this infection in young adults [8] and children [5, 10].

The sex ratio of our patients was 1.6. This male predominance of bilharziasis has been objectified by several studies carried out in Africa [12–14] and is explained by professional exposure during farming and fishing. However, according to Guiguen et al., women are more exposed to schistosomiasis in areas that do not have access to running water because in such case, women are in contact with infesting water during domestic work [4].

Hematuria was the finding in all patients and was associated with difficulty passing urine in two patients. El-Badawi had also objectified hematuria as the main symptom (73.3%) associated with burning sensation when voiding (66.3%), dysuria (59.3%), frequency (58.1%), suprapubic pain (50%), pyuria (44.2%), or renal colic (30.2%). Renal colic was explained by the obstruction of the ureteral meatus by the mass or a stenosing fibrosis of the ureter [8].

Abdel-Salam and Ehsan, on the other hand, reported difficulty passing urine as a predominant symptom in patients with a granulomatous polyp and in more than 50% of patients with a fibrocalcic polyp [9]. The other symptoms he described were hematuria and pyuria [9].

A urine analysis was performed in two of our patients and showed bilharzia eggs in one of them. According to Abdel-Salam, the presence of viable eggs is very sensitive for the diagnosis of granulomatous polyps (70%) and less sensitive in fibrocalcic polyps (16.5%) [9].

We had reported 38.5% of granulomatous polyps and 61.5% of fibrocalcic polyps. El-Badawi [8], on the other hand, found a predominance of granulomatous polyps (60.5%), while Abdel-Salam and Ehsan [9] showed an equal proportion of granulomatous and fibrocalcic polyps.

In the area of schistosomiasis endemic, large granulomatous polyps can be confused with squamous cell carcinoma of the bladder. But they can be differentiated by their typical location at the trigone and the paraureteral region, their multiple numbers, the pedunculated aspect, red appearance, and the tendency to bleed when pressed by the cystoscope. The regression of such lesion after antibilharzian treatment has a great diagnostic value [8].

Fibrocalcic polyps can be confused with bladder stones, but they do not move freely like stones with the filling of the bladder or when pushed by the cystoscope. They are pedunculated, dull yellowish in color, and occur in patients over 20 years of age [8].

Three of the granulomatous polyps (60%) and 4 of the fibrocalcic polyps (50%) were associated with semolina grain appearance. El-Badawi [8] reported bilharzian lesions (bruises and semolina grains appearance) associated with granulomatous polyps in the majority of cases (96%). He also observed that fibrocalcic and villous polyps were associated with lesions of chronic bilharzian cystitis in the majority of cases and active lesions in a minority of cases (8.8%). Pinto et al. had also observed lesions of active bilharziasis associated with granulomatous polyps [5, 10].

In our series, patient who had pathologic features of dysplasia and metaplasia were lost to follow-up. Regular endoscopic monitoring of these patients would have made it possible to diagnose a possible evolution towards squamous cell carcinoma. Indeed, according to Berry et al. [15], the genesis of carcinoma follows the sequence hyperplasia-metaplasia-dysplasia-carcinoma. Amin et al. [16] also reported the diagnosis of squamous cell carcinoma of the bladder following bilharzioma.

The rapid regression of granulomatous polyps under antibilharzian treatment limited the indications for surgery. Surgery can be performed if medical treatment fails or when residual polyps obstruct the urethral orifices or bladder neck. Small, nonobstructing fibrocalcic polyps without further damage do not require treatment, unless there is evidence of an active schistosomiasis. The villous polyps must be completely excised to allow a careful histological examination [8].

Pinto et al. have successfully treated granulomatous polyps with medical treatment [5, 10].

We have associated transurethral resection of the bladder with praziquantel in all our patients. A good characterization of the lesions at cystoscopy would have allowed us to evoke the diagnosis of bladder bilharzioma and to reserve surgery for therapeutic failures. Indeed, the treatment of uncomplicated bladder bilharziomas falls under the antibilharzian treatment. Praziquantel at a dose of 40 mg/kg is recommended. Transurethral resection of the bladder and bladder biopsy are indicated for residual masses or when the endoscopic diagnosis of bilharzioma is not obvious [6].

Our study shows the importance of pathology in the diagnosis of bladder schistosomiasis lesions. It is important in our region to consider the diagnosis in spite of the compelling need to consider the possibility of neoplasm. While we acknowledge a limitation related to the small sample size, our study shed light in the endoscopic and pathologic feature of bilharzioma in the setting of schistosomiasis endemic.

## 5. Conclusion

Bladder bilharziomas pose the problem of differential diagnosis with squamous cell carcinoma and bladder stones in areas of bilharzian endemic. Nevertheless, a clever exploration with cystoscopy would allow to evoke the diagnosis and to initiate the antibilharzian treatment sometimes supplemented by an endoscopic resection of the bladder lesion.

## Figures and Tables

**Table 1:** Clinical information of the patients.

	Age	Sex	Residence	Notion of swimming in fresh water	Clinical sign
Patient 1	42	F	Fouta	Yes	Hematuria
Patient 2	24	F	Fouta	Yes	Hematuria
Patient 3	16	F	Fouta	Yes	Hematuria
Patient 4	17	F	Dakar	Unspecified	Hematuria
Patient 5	17	F	Fouta	Yes	Hematuria + dysuria
Patient 6	45	M	Dakar	Yes	Hematuria
Patient 7	23	M	Mauritanie	Unspecified	Hematuria
Patient 8	32	M	Fouta	Yes	Hematuria
Patient 9	23	M	Fouta	Yes	Hematuria
Patient 10	12	M	Fouta	Yes	Hematuria
Patient 11	26	M	Fouta	Yes	Hematuria + dysuria
Patient 12	48	M	Fouta	Yes	Hematuria
Patient 13	26	M	Fouta	Yes	Hematuria

**Table 2:** Distribution of polyps according to their characteristics.

Characteristics of bladder polyps	Granulomatous polyps	Fibrocalcium polyps	Polyps villous
Seat of polyps			
Right perimeatic	3	2	0
Left perimeatic	2	1	0
Trigone	0	0	0
Posterior side	0	1	0
Dome	0	4	0

Number of polyps			
Unique	2	6	0
Multiple	3	2	0

## Data Availability

All the information and data for this manuscript is available in the main text and uploaded as supporting documents. Name and identity of the patient has been concealed for confidentiality.
